# Combined Effects of Elevated *p*CO_2_ and Warming Facilitate Cyanophage Infections

**DOI:** 10.3389/fmicb.2017.01096

**Published:** 2017-06-13

**Authors:** Kai Cheng, Dedmer B. Van de Waal, Xiao Ying Niu, Yi Jun Zhao

**Affiliations:** ^1^Hubei Key Laboratory of Ecological Restoration for River-Lakes and Algal Utilization, College of Resources and Environmental Engineering, Hubei University of TechnologyWuhan, China; ^2^College of Life Science, Central China Normal UniversityWuhan, China; ^3^Department of Aquatic Ecology, Netherlands Institute of EcologyWageningen, Netherlands

**Keywords:** climate change, cyanobacterial virus, infectivity, one-step growth curve, EOP, common garden experiment

## Abstract

Elevated *p*CO_2_ and warming are generally expected to influence cyanobacterial growth, and may promote the formation of blooms. Yet, both climate change factors may also influence cyanobacterial mortality by favoring pathogens, such as viruses, which will depend on the ability of the host to adapt. To test this hypothesis, we grew *Plectonema boryanum* IU597 under two temperature (25 and 29°C) and two *p*CO_2_ (400 and 800 μatm) conditions for 1 year, after which all treatments were re-exposed to control conditions for a period of 3 weeks. At several time points during the 1 year period, and upon re-exposure, we measured various infection characteristics of it associated cyanophage PP, including the burst size, latent period, lytic cycle and the efficiency of plaquing (EOP). As expected, elevated *p*CO_2_ promoted growth of *P. boryanum* equally over the 1 year period, but warming did not. Burst size increased in the warm treatment, but decreased in both the elevated *p*CO_2_ and combined treatment. The latent period and lytic cycle both became shorter in the elevated *p*CO_2_ and higher temperature treatment, and were further reduced by the combined effect of both factors. Efficiency of plaquing (EOP) decreased in the elevated *p*CO_2_ treatment, increased in the warm treatment, and increased even stronger in the combined treatment. These findings indicate that elevated *p*CO_2_ enhanced the effect of warming, thereby further promoting the virus infection rate. The re-exposure experiments demonstrate adaptation of the host leading to higher biomass build-up with elevated *p*CO_2_ over the experimental period, and lower performance upon re-exposure to control conditions. Similarly, virus burst size and EOP increased when given warm adapted host, but were lower as compared to the control when the host was re-exposed to control conditions. Our results demonstrate that adaptation but particularly physiological acclimation to climate change conditions favored viral infections, while limited host plasticity and slow adaptation after re-exposure to control conditions impeded host biomass build-up and viral infections.

## Introduction

The climate is changing at an unprecedented rate, and atmospheric *p*CO_2_ is predicted to have doubled from 400 μatm today to 800 μatm by the end of this century. Elevated *p*CO_2_, together with increases in concentrations of other greenhouse gases, is predicted to enhance the average global temperature by up to 4.8°C (Stocker et al., 2013). Phytoplankton play a key role in global carbon cycling, and contribute to approximately 50% of the CO_2_ fixed by the entire biosphere (Field et al., 1998). Photosynthesis and carbon fixation are expected to increase further with elevated *p*CO_2_ in both marine (Hein and Sand-Jensen, 1997) as well as freshwater ecosystems (Verspagen et al., 2014a). Elevated *p*CO_2_ may particularly favor photosynthesis and growth of cyanobacteria, as they feature among the lowest affinity enzymes for carbon fixation, i.e., ribulose-1,5-bisphosphate carboxylase/oxygenase (RubisCO) type 1D (Badger et al., 2006; Raven et al., 2008). Cyanobacterial blooms are furthermore associated to warm conditions, and it is thus this phytoplankton group that may be particularly favored by climate change (Paerl and Huisman, 2009; Carey et al., 2012; Kosten et al., 2012; O'Neil et al., 2012). The responses of cyanobacteria to climate change will also depend on their adaptive abilities (Walworth et al., 2016). Earlier studies, however, did not find evidence for evolutionary change toward elevated *p*CO_2_ in two freshwater cyanobacteria species (Low-Décarie et al., 2013), while data on warming effects seems currently lacking.

The success of phytoplankton species in future waters will not only depend on their growth and abilities to adapt, but also on mortality factors, notably their pathogens, such as viruses (Suttle, 2007). Viruses control the structure of entire phytoplankton communities as they play a key role in regulating host population densities, the cycling of carbon and nutrients (Fuhrman, 1999; Weinbauer, 2004; Jover et al., 2014), as well as in host evolution via horizontal gene transfer (Thompson et al., 2011) and genotype selection (Suttle, 2007). Cyanophages (i.e., cyanobacteria viruses) have been reported for a wide range of species, including notorious toxin producers, such as *Nodularia, Dolichospermum* (formerly *Anabaena*; Wacklin et al., 2009), *Planktothrix, Aphanizomenon* and *Microcystis* (Jenkins and Hayes, 2006; Yoshida et al., 2006; Gao et al., 2012; Ou et al., 2015; Sulcius et al., 2015), and were reported to remove up to 97% of the potential filamentous cyanobacterial production in a shallow eutrophic lake (Tijdens et al., 2008).

Climate change may alter virus-host interactions, and will thereby affect the functioning of aquatic food-webs (Danovaro et al., 2011). Elevated *p*CO_2_ and warming were reported to have a range of effects on the viral infection of phytoplankton. For instance, elevated *p*CO_2_ may enhance (Zhou et al., 2015) or inhibit (Larsen et al., 2008) viral infections, while some studies also reported a lack of effect (Maat et al., 2014). With respect to infection characteristics, burst size was shown to increase (Carreira et al., 2013; Zhou et al., 2015) or decrease (Traving et al., 2014) with elevated *p*CO_2_, and also the latent period did not show unambiguous results even within a single virus-host system (Traving et al., 2014). Comparably, warming was shown to cause an increase in infections, as indicated by enhanced virus concentrations (Honjo et al., 2007; Chu et al., 2011; Mankiewicz-Boczek et al., 2016), but may also reduce infections, depending on the specific virus (Tomaru et al., 2014), as well as on temperature dependent host resistance (Kendrick et al., 2014).

The effects of elevated *p*CO_2_ and warming on virus-host interactions have thus been investigated in various host species from distinct ecosystems, yet results remain ambiguous and only little is known about their combined effects over different temporal scales. Elevated *p*CO_2_ combined with warming were shown to affect key phytoplankton traits, such as enhanced growth rates with reduced cell sizes (Fiorini et al., 2011), increased carbon:nutrient ratios (Fu et al., 2007, 2008; Muller et al., 2014; Verspagen et al., 2014b; Paul et al., 2015), and enhanced primary production (Holding et al., 2015). Viruses rely on their host cells for reproduction, and the success of an infection thus strongly depends on the physiological status of a host (Mojica and Brussaard, 2014). In other words, conditions enhancing growth of a host, may also promote growth of their virus. Consequently, we predict that elevated *p*CO_2_ and warming will facilitate viral infections of a cyanobacterial host, and that their combined effect will be synergistic. Moreover, with a generation time of about 2 days we expect that host cells will adapt over a 1 year period under the imposed conditions, as this covers over 150 generations, which will further enhance viral infections. To test these hypotheses, we exposed the freshwater cyanobacterium *Plectonema boryanum* to elevated *p*CO_2_, warming and a combination of both, and followed key physiological traits of the host and its cyanophage over 1 year period, as well as after re-exposure to control conditions. More specifically, traits of the host included growth rate, cell size and chlorophyll-a contents, which are key parameters indicating host fitness. For the cyanophage, we assessed the latent period (the period from host infection to initial phage release), the lytic cycle (the period from host infection to complete phage release), and burst size (the average number of phages released from a single infected host cell). Moreover, we tested the efficiency of plaquing (EOP), which is proportion of cyanophages that can successfully infect to host in 1 h.

## Materials and methods

### Cyanophage and cyanobacteria

Cyanophage PP is a podovirus with a linear, double-stranded DNA genome that has been frequently detected at high levels in many eutrophic lakes in China (Cheng et al., 2007). The experiments were performed with a cyanophage PP lysate containing >10^8^ plaque forming units (PFU) mL^−1^ that was stored at 4°C. The cyanobacterium *P. boryanum* IU597, obtained from the FACHB-collection, Wuhan City, China, was cultivated in AA medium (Allen and Arnon, 1955), with 30 μmol photons m^−2^ s^−1^ white light (Philips, Lifemax TLD 36W/865, China) and a 12:12 h light:dark cycle. Experiments were performed in 200 mL growth medium in triplicate, and incubated in self-made chambers at different combinations of *p*CO_2_ and temperatures: 400 μatm *p*CO_2_ and 25°C (control), 400 μatm *p*CO_2_, and 29°C (warm treatment), 800 μatm *p*CO_2_ and 25°C (elevated *p*CO_2_ treatment), and 800 μatm *p*CO_2_ and 29°C (combined treatment). Additional CO_2_ for the elevated *p*CO_2_ treatment was supplied to the headspace, and was measured using a Telaire 7001 CO_2_ sensor (USA). Initial pH reflected the CO_2_ treatments with values of 6.72, 6.71, 6.50, and 6.52, for the control, warm, elevated *p*CO_2_ and combined treatment, respectively.

To prevent transient effects of elevated *p*CO_2_ and warming on host growth, *P. boryarum* was conditioned to the above treatments for 1 year during which the cultures were re-inoculated by diluting the cell density to 2.0 × 10^6^ cells mL^−1^ with AA medium every 15 days. Then, the cultures in each condition were all re-exposed to the control condition for a period of 3 weeks (i.e., a common garden experiment). Host growth and viral infectivity were tested at 6, 9, and 1 year within 1 year and also after the re-exposure to control conditions. Cells were counted and cell size was measured by microscopy (E600, Nikon, Japan) using a hemocytometer, and the chlorophyll-a (chl-a) concentration was measured by acetone extraction (Chen et al., 2005) at day 7. The growth rate of the host (μ) was calculated assuming exponential growth based on cell numbers at the start of the experiment and at day 7 according to:

$$\begin{array}{l} \mu =\frac{ln\left(X_{7}\right)-ln\left(X_{0}\right)}{t_{7}-t_{0}} \end{array}$$

Where *X*_7_ and *X*_0_ represent *P. boryarum* cell densities at day 7 (i.e., *t*_7_) and day 0 (i.e., *t*_0_), respectively. All experiments started with the same initial cell densities (i.e., *t*_0_) of 2.0 × 10^7^ cells mL^−1^ by dilution with AA medium.

*P. boryarum* cultures were subsequently used to assess key viral traits, including latent period, lytic cycle, and the average burst size with one-step growth assays, and EOP with plaque assays.

### One-step growth assay

The one-step growth assays were performed to assess the latent period, lytic cycle, and the average burst size. The effect of initial multiplicity of infection (MOI, i.e., the number of phages per host cell) on the latent period and burst size is specific for any particular virus-host system (Sulcius et al., 2015). The MOI is typically set at about 1 phage per host cell for uni- or bicellular hosts to avoid host cell infection by more than one phage, and also to ensure that most of the cells will be infected in only one step (Bratbak et al., 1998; Yoshida et al., 2006). However, *P. boryanum* used in our study grows in trichomes, consisting of up to 800 cells. Assuming a viral adsorption fraction of 10% (i.e., fraction of viruses that adsorp to a host) and a burst size of 100 PFU cell^−1^, an initial MOI of more than 0.125 × 10^−3^ would lead to multiple infections after a first round of lysis. Thus, in this one-step growth assay, host cell suspensions (50 mL) were mixed with a cyanophage lysate at a sufficiently low MOI of 0.1 × 10^−4^. After 30 min of steady incubation to allow adsorption, the mixtures were centrifuged at 10,000 × *g* for 10 min at the corresponding culture temperature. The pellets were collected, washed twice in AA medium, and then resuspended in 50 mL of AA medium and incubated in the chambers described above. For the plaque assays (Suttle, 1993), 0.1 and 1 mL samples from these resuspended cultures were plated at 0, 60, 90, 120, 180, 240, 300, 360, and 390 min. After constructing one-step growth curves (Supplementary Figure 1) with cyanophage titres at each time point relative to those at *t*_0_, the latent period, lytic cycle and average burst size were determined using a modified Gompertz sigmoid growth function (Zwietering et al., 1990) including the addition of a normalized initial PFU of 1:

$$\begin{array}{l} y=B\times exp\left(-exp\left(\frac{r_{m}\;\times e}{B}\left(\lambda -t\right)+1\right)\right)+1 \end{array}$$

where *y* indicates the titre at time *t, B* the burst size (i.e., the maximum relative PFU), *r*_*m*_ the maximum infection rate, *e* the mathematical constant (i.e., 2.718), and λ is the latent period indicated by the point on the x-axis where the slope from the maximum increase crosses *y* = 1. Fits were performed using least square fitting with the Microsoft Excel 2013 Solver GRG nonlinear fitting procedure with multistart of population size of 200. The lytic cycle was estimated based on the relative PFU data as the time when the burst size B was reached.

### EOP assay

For the EOP assay, host cell suspensions were sampled and a cyanophage lysate was added with a MOI of 0.1 × 10^−3^. The titer of the mixture was recorded as *P*_0_. Such a low MOI should prevent multiple phages attaching to a single trichome, which consisted of 120 cells on average with exceptions of up to 800 cells. These mixtures were subsequently incubated for 1 h for viral adsorption, without shaking, in the chambers described above. To assess the number of infected hosts at the end of the assay (*P*_1_), 1.5 mL of sample was centrifuged at 12,000 × *g* for 10 min at the corresponding culture temperature. The supernatant containing free cyanophages was removed, and the titres of the pellets containing infected cyanobacteria were subsequently determined by plaque assay (Suttle, 1993). The EOP over a 1 h period was calculated as *P*_1_/*P*_0_.

### Statistical analysis

Data was log transformed before statistical analysis to improve equality of variances. Normality and equality of variances were confirmed using the Kolmogorov-Smirnov test and Levene's test, respectively. Significant differences between treatments were tested using a one-way ANOVA, and followed by *post-hoc* comparison of the means using S-N-K test if normality was confirmed, otherwise the Games-Howell test was applied. Statistics were performing with SPSS Statistics 17.0 (IBM Inc., USA), the figures were made by GraphPad Prism 5.01 (GraphPad Inc., USA), and all values represent means (*n* = 3) ± standard deviation (SD). Significant differences between treatments were indicated by different lowercase letters.

## Results

**Figure 1:**
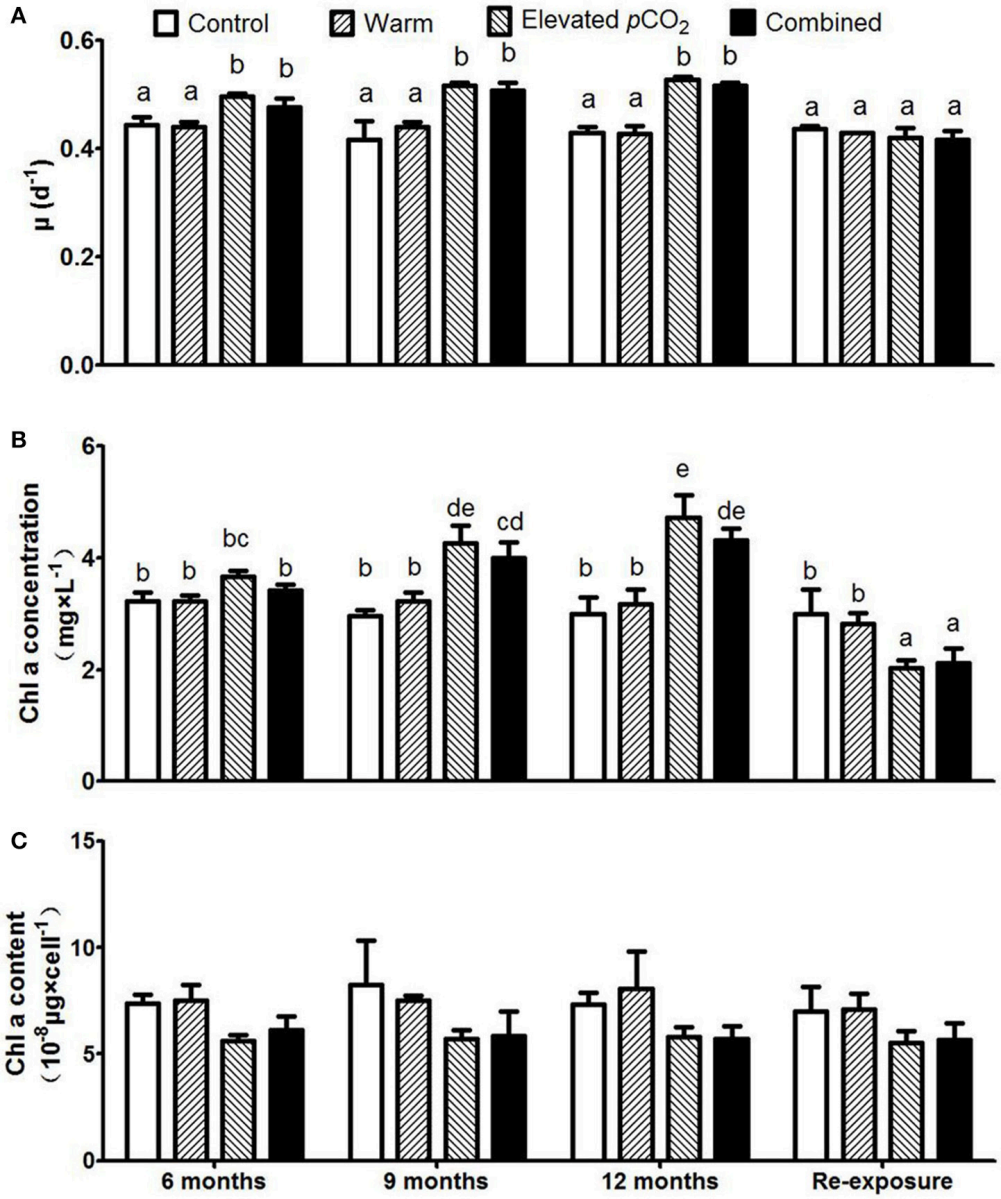
Responses of *Plectonema boryanum* to elevated *p*CO_2_, temperature and a combination of both treatments with growth rate **(A)**, Chl-a concentration **(B)**, and Chl-a content per cell **(C)**. Bars show mean ± SD (*n* = 3). Different lowercase letters indicate significant differences between treatments (one-way ANOVA, *P* < 0.05).

The growth rates of *P. boryanum* were significantly higher in the elevated *p*CO_2_ and combined effect treatments as compared to the control conditions (Figure 1A, *P* < 0.05), and were in accordance with the observed effects on Chl-a concentrations (Figure 1B). While growth rate remained unaltered during the course of 1 year in these two treatments, host cell density (Supplementary Figure 2) and Chl-a concentrations showed a gradual increase (*P* < 0.05). Cellular Chl-a contents did not significantly change with warming, elevated *p*CO_2_, nor their combined effect, though tended to be higher in the warm treatment compared to the elevated *p*CO_2_ and combined effect treatment (Figure 1C). After re-exposure to control conditions, Chl-a concentrations in the elevated *p*CO_2_ and combined treatment turned to lower than the control (*P* < 0.05).

**Figure 2:**
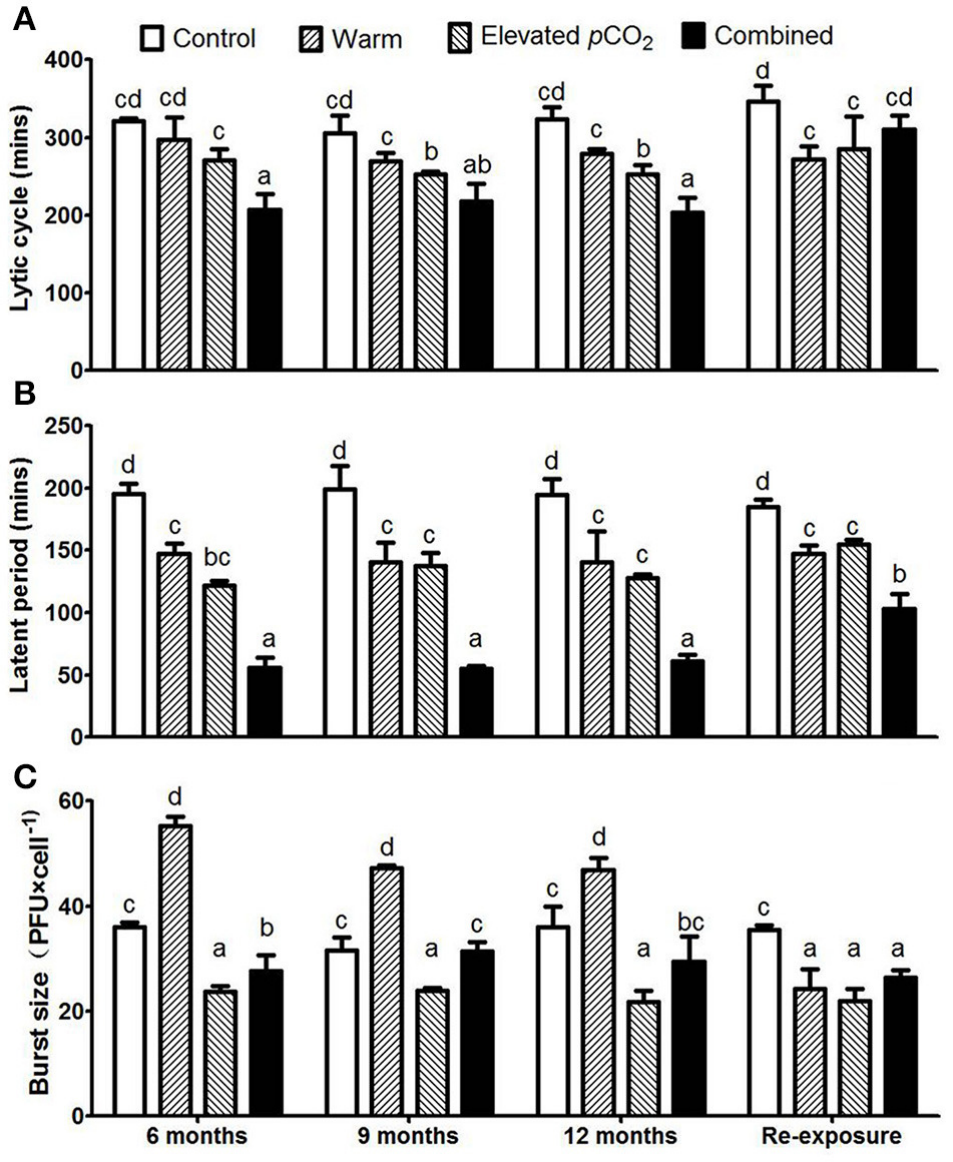
The dynamic parameters of one step growth curves for each treatment with lytic cycle **(A)**, latent period **(B)**, and average burst size **(C)**. Bars show mean ± SD (*n* = 3). Different lowercase letters indicate significant differences between treatments (one-way ANOVA, *P* < 0.05).

The lytic cycle, latent period and burst size remained largely unaltered over the course of the 1 year period (Figure 2). However, the lytic cycle was reduced from 316 ± 10 min in the control to 282 ± 14 min and 256 ± 11 min for warming and elevated *p*CO_2_ treatment, respectively (Figure 2A, *P* < 0.05). When both factors were combined, the lytic cycle further shortened down to 209 ± 8 min. The lytic cycle includes the latent period, which was strongly reduced from 196 ± 3 min in the control, down to 143 ± 4, 129 ± 8, and 57 ± 3 min in the warming, elevated *p*CO_2_ and combined effect treatment, respectively (Figure 2B; *P* < 0.05). The difference between the lytic cycle and latent period (Δt) did not differ between the treatments (Supplementary Figure 3), thus indicating that the shorter lytic cycles were a result of a shorter latent period. Upon re-exposure to control conditions, the lytic cycle and latent period remained lower for warming and elevated *p*CO_2_ treatments as compared to the control (Figures 2A,B; *P* < 0.05), while the lytic cycle and latent period turned to increase in the combined treatment as compared to before re-exposure (Figures 2A,B; *P* < 0.05).

Burst size increased from 35 ± 3 PFU cell^−1^ to 50 ± 5 PFU cell^−1^ in response to warming (*P* < 0.05), whereas it decreased to 23 ± 1 PFU cell^−1^ with elevated *p*CO_2_ (Figure 2C; *P* < 0.05). Both factors seemed to counteract each other when combined, as the burst size of the combined effect treatment was 29 ± 2 PFU cell^−1^ and thus demonstrated no significant difference to the control. The combined effect of elevated *p*CO_2_ and warming on burst size and latent period seems additive rather than interactive. After re-exposure to the control conditions, the burst size was lower as compared to the control for all climate change factors (Figure 2C; *P* < 0.05).

**Figure 3:**
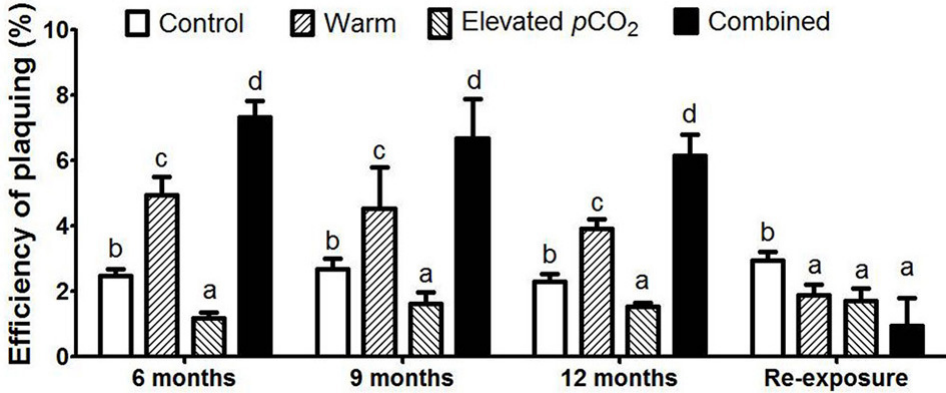
The EOP for each treatment. Bars show mean ± SD (*n* = 3). Different lowercase letters indicate significant differences between treatments (one-way ANOVA, *P* < 0.05).

The EOP was not influenced by the duration of exposure, but increased from 2.5 ± 0.2% in the control to 4.5 ± 0.5% in the warm treatment, whereas it decreased down to 1.5 ± 0.2% in the elevated *p*CO_2_ treatment (Figure 3). Together, warming and elevated *p*CO_2_ resulted in an EOP of 6.7 ± 0.6%, indicating a clear interactive effect of elevated *p*CO_2_ and warming on EOP (*P* < 0.05). In other words, the increase in EOP with warming was further enhanced with elevated *p*CO_2_, and both factors thus synergistically favored infection by cyanophage PP. After re-exposure to control conditions, EOP responded similarly as the burst size, with a significant decrease as compared to the control in all climate change treatments (Figure 3; *P* < 0.05).

## Discussion

### Physiological acclimation and evolutionary adaptation of host and virus

The growth rates of *P. boryanum* ranged from 0.42 to 0.53 in this work, and it was lower than *P. boryarum* UTEX 485 (Miśkiewicz et al., 2000), but higher than *P. boryarum* AUCC 143 (Prasad et al., 2005). Cyanobacteria possess RubisCO 1D with a low affinity for CO_2_ (Badger et al., 2006), and their growth was thus expected to be favored with elevated *p*CO_2_. Indeed, growth and biomass build-up (as indicated by Chl-a concentration) of *P. boryanum* showed a consistent increase with elevated *p*CO_2_ and with elevated *p*CO_2_ combined with warming over the 1 year period. Yet, after a 3 week period of re-exposure to control conditions, growth was similar to the control conditions while biomass build-up was lower for cells grown at both conditions. The observed gradual increase in biomass build-up over the 1 year period may indicate adaptation to elevated *p*CO_2_ levels. Moreover, these results suggest that this adaptive response regarding biomass build-up becomes unfavorable when exposed again to low CO_2_ availabilities. These findings are in contrast to earlier studies that did not reveal evolutionary adaptation in cyanobacteria (Low-Décarie et al., 2013), but seems comparable to observations on the green alga *Chlamydomonas*, showing reduced growth rates upon a short re-exposure to ambient *p*CO_2_ conditions after long-term elevated *p*CO_2_ exposure (Collins and Bell, 2004).

Adaptation to elevated *p*CO_2_ thus seem favorable for *Plectonema* as it could reach higher biomass build-up, but this will only occur if CO_2_ levels will remain high for a sufficient number of generations. In our experiment, *Plectonema* was exposed to the climate change factors for over 150 generations, which appeared sufficiently long to allow adaptive responses. CO_2_ concentrations in productive freshwater systems may vary substantially at short time scales as result of uptake of CO_2_ via photosynthesis (Verspagen et al., 2014a). Under such conditions, adaptation is likely not occurring and the success of a species will largely depend on the physiological acclimation (i.e., phenotypic plasticity). In less productive waters, CO_2_ concentrations may become high and remain more stable. Under such conditions, adaptive responses are more likely to occur. In contrast to our expectations, warming did not cause an increase in growth or biomass build-up in our study. This is surprising, as cyanobacteria tend to have higher growth optima with a generally broad temperature range (Lürling et al., 2013). An earlier study with *P. boryanum* did show an increase in growth rate and biomass build-up after 16 h of warming from 15 to 29°C (Miśkiewicz et al., 2000). Our control conditions were performed at 25°C, which may have already been the growth optimum for this species. Although a warming of 4°C is likely to affect *P. boryanum* metabolism, it did not result in differences in growth rate or biomass build-up. Moreover, it seems that warming did not alter the response of the host to elevated *p*CO_2_. Impacts of warming will thus not only depend on the extent by which temperature increases, but also on the temperature range where these changes occur relative to an organism's growth optimum.

Although virus infection dynamics changed with climate change factors, the observed responses remained largely unaltered over the 1 year period (i.e., the response after 1 year was similar to the response after 6 months). This suggests that virus infection dynamics were insensitive to the changes in host fitness, or that the changes in host fitness over the course of 1 year were too small relative to the differences between the treatments. Consequently, the main response of virus infections follows host physiological acclimation rather than adaptation. Interestingly, burst size and infection efficiency (i.e., EOP) of the virus showed a completely reversed response when provided with host cells that were re-exposud to control conditions after having grown in the warm and/or combined treatment. This may indicate that host cells were adapted to the warm and combined conditions, and that upon re-exposure the physiological acclimation to control conditions reduced the virus burst size and infection efficiency. Thus, host adaptation but particularly physiological acclimation to climate change factors are responsible for the observed shifts in viral infectivity.

### Infection dynamics

Our result show various CO_2_ and temperature effects on cyanophage infection characteristics, and both factors may promote but also impede infections. For instance, elevated *p*CO_2_ led to a shorter latent period but also a reduced burst size. Thus, faster infection cycles are accompanied by a reduced production of offspring phages. Such a trade-off has also been reported earlier for a cyanophage-*Synechococcus* system where a shorter latent period in response to a lower pH was accompanied by a reduced burst size (Traving et al., 2014). Under elevated *p*CO_2_, growth rate of the host increased which may have led to a shorter latent period and consequently result in a smaller burst size, as there is less time for phage production (Gnezda-Meijer et al., 2006). Impacts of elevated *p*CO_2_ on the burst size of phytoplankton viruses, however, do not seem unambiguous. For instance, burst size of an *Emiliania huxleyi* virus was shown to increase (Larsen et al., 2008) or decrease (Carreira et al., 2013), while no *p*CO_2_ dependent change in burst size was observed for a *Micromonas pusilla* virus (Maat et al., 2014), though in the latter the host was P-limited. Warming alone caused a shorter latent period along with higher burst size, which was also reported for the *Lactococcal* phage P008 (Mueller-Merbach et al., 2007) and the *Streptococcus* phage phi18 (Sanders and Klaenhammer, 1984). This suggests that warming may favor phage development, possibly by stimulating enzymes involved in phage production (Chu et al., 2011), and/or inhibit cellular defense mechanisms against phage infection (see also below) (Sanders and Klaenhammer, 1984; Durmaz and Klaenhammer, 2007). The combined effect of warming and elevated *p*CO_2_ on latent period and burst size were additive, resulting in a further shortening of the latent period but a reduced overall effect on burst size.

### Infection efficiency

Phage infection generally depends on (1) the contact rate between the phage and host, and (2) phage resistance mechanisms by the host cell. The rate of contact is influenced by the concentration of host and virus, host cell size, temperature, and viscosity of the water (Murray and Jackson, 1992). Indeed, virus infections seem generally density dependent (Murray and Jackson, 1992), and larger host cells were shown to provide a greater surface area for contact (Hadas et al., 1997). In the EOP assay, host, cyanophage concentration and the cell size were similar between treatments (Supplementary Figure 4), and these factors thus cannot explain differences in EOP. In the warm treatments, water temperature was increased from 25 to 29°C, with a consequent decrease in water viscosity from 0.8937 mPa × s to 0.8180 mPa × s. This would lead to an increased contact rate by 10.7% (Murray and Jackson, 1992). Thus, the observed increase in EOP in the warm and combined effect treatments could, at least partially, be explained by a temperature dependent decrease in water viscosity.

Efficiency of plaquing (EOP) was more than 50% higher in the warm treatment compared to the control, which is comparable to earlier reports on plaquing efficiency with a cyanophage—*Dolichospermum* system (Currier and Wolk, 1979). This suggests that warming may impede phage resistance mechanisms by the host cell, including (1) blocking of adsorption, (2) blocking of phage DNA injection, (3) DNA restriction/modification (R/M), and (4) abortive infection (Abi), which may be the main reason for the higher EOP. Both higher pH (caused by lower *p*CO_2_) and higher temperature (Benedi et al., 1991) can lead to a higher lipopolysaccharide content on the cell surface that may act as receptor for cyanophage adsorption (Samimi and Drews, 1978). Thus, elevated *p*CO_2_, resulting in lower pH, may reduce adsorption, while warming may enhance adsorption via changes in the cell surface lipopolysaccharides. Also DNA injection was shown to be influenced by both pH and temperature (Kumar Sarkar et al., 2006), and an increase in temperature was furthermore shown to enhance DNA injection (Labedan and Goldberg, 1979). Higher temperatures may also promote the DNA ejection rate, which could be explained by a temperature induced conformational change in the tail pore-forming proteins allowing phage DNA to pass through faster (Lof et al., 2007). R/M systems are widely distributed in bacteria as well as in unicellular and filamentous cyanobacteria (Szekeres et al., 1983; Zhao et al., 2006; Stucken et al., 2013). The activity of R/M systems mainly depends on various restriction endonucleases to digest phage DNA, with distinct optimal digestion temperatures and pH (Sanders and Klaenhammer, 1984; Guimont et al., 1993; Dempsey et al., 2005). Lastly, Abi systems can cause a disruption of phage development after early phage gene expression, resulting in a decrease in death of the infected cells and/or a reduction in phage burst size (Emond et al., 1997; Tangney and Fitzgerald, 2002). The activity of Abi genes may be up- or down-regulated by temperature (Tangney and Fitzgerald, 2002; Bidnenko et al., 2009), but there is no report on the influence of *p*CO_2_ or pH on Abi gene activity.

Elevated *p*CO_2_ magnified the positive effect of warming on EOP, even though elevated *p*CO_2_ alone caused a decrease in EOP. These surprising interactive effects suggest a dependency of infection characteristics on both temperature and *p*CO_2_. Dissociation of the cell puncturing complex of phages is essential for phage DNA injection, and was shown to be controlled by both pH and temperature (Kumar Sarkar et al., 2006). Specifically, a decreasing pH slowed down the dissociation of a protein complex that is involved in host resistance (i.e., (gp5^*^)_3_(gp5C)_3_) when temperature was 0°C, while the dissociation of the same complex was accelerated by a decreasing pH at 20°C. These results suggest that the optimal pH for phage DNA injection could vary strongly with temperature. Warming and elevated *p*CO_2_, together with associated changes in pH, may thus affect the resistance of a host to phage infection. Future studies should further elucidate the complex interplay between climate change effects on phage associated traits and host defense mechanisms.

## Conclusions

In conclusion, the mechanisms responsible for the combined effects of elevated *p*CO_2_ and elevated temperature on the infectivity of cyanophage are complex and poorly understood. Although we observed host adaptation to the climate change factors, the strongest virus responses seem a result of host physiological acclimation. Warming was shown to increase EOP, as well as reduce the latent period and increase burst size, while elevated *p*CO_2_ reduced EOP, latent period and burst size. Thus, warming seems to generally promote phage infections, while elevated *p*CO_2_ differentially affects various components of phage infections. The observed effects of both climate change factors are additive, except for EOP for which the increase with warming was promoted by elevated *p*CO_2_. Thus, the combined influence of warming and elevated *p*CO_2_ may have a profound influence on the virus-host dynamics with climate change. More specifically, although climate change may promote cyanobacteria growth, their phages may also be facilitated and thereby pose a stronger control on cyanobacterial bloom formation.

## Author contributions

KC and YJZ had the idea of the experiment. KC was responsible for conducting the experiment together with XYN, and wrote the first manuscript draft with input from all co-authors. DBVDW and KC ran data analyses and wrote the final version of the manuscript.

### Conflict of interest statement

The authors declare that the research was conducted in the absence of any commercial or financial relationships that could be construed as a potential conflict of interest.
