# Supplementary material for: Combined Effects of Elevated pCO2 and Warming Facilitate Cyanophage Infections
Source: Front Microbiol. 2017 Jun 13;8:1096. doi: 10.3389/fmicb.2017.01096 (PMC5468398; doi:10.3389/fmicb.2017.01096)
Supplement: Supplementary file 1 [file Image1.pdf]

Supplementary Material

Combined Effects of Elevated pCO_2 and Warming Facilitate Cyanophage Infections

Cheng Kai[1,3]*, Dedmer B. Van de Waal[2], Niu Xiaoying[3] and Zhao Yijun[1]*

*** Correspondence:** Cheng Kai: chengkaicn@163.com

1 Supplementary Figures

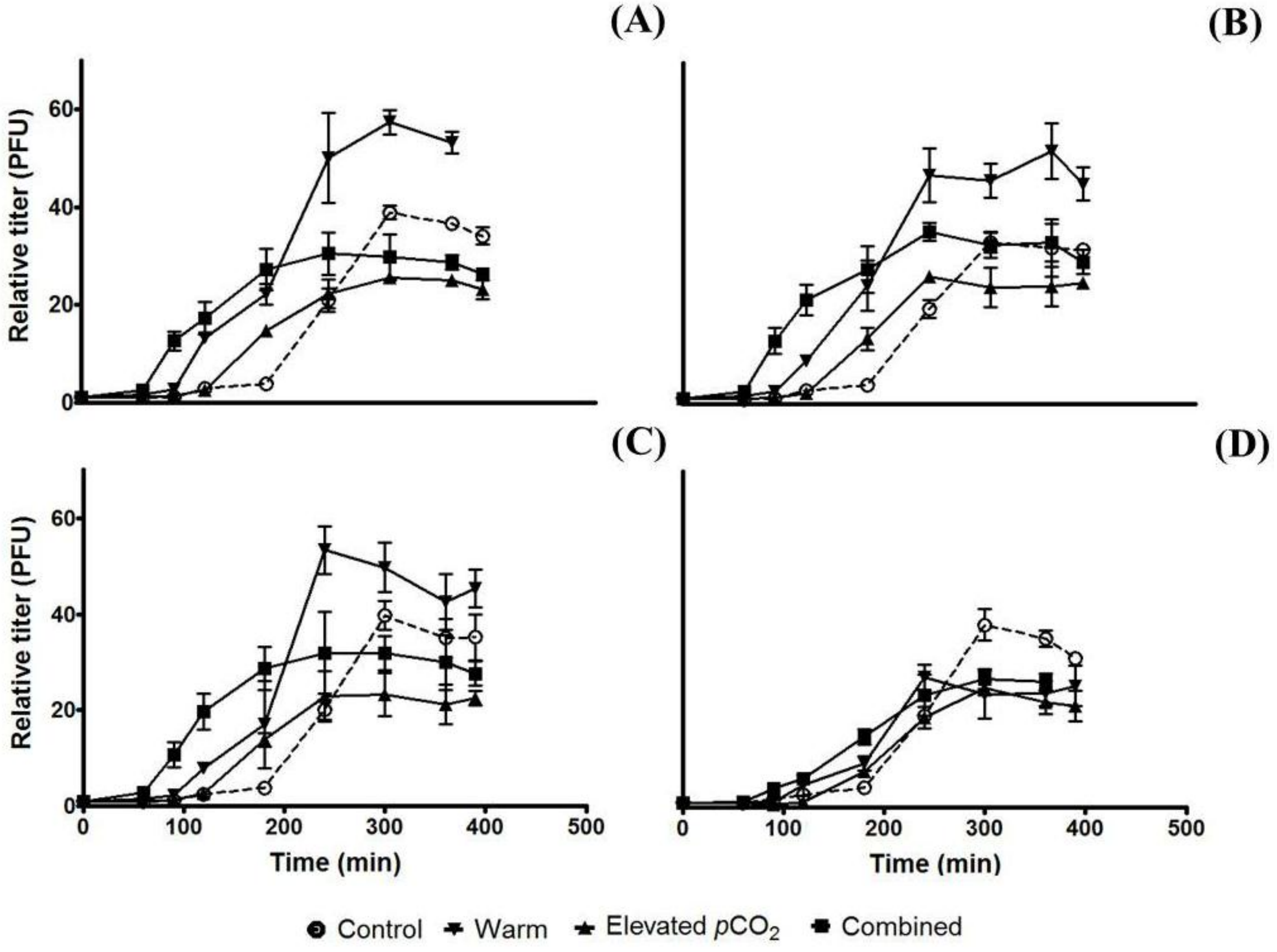

Supplementary Figure 1. One-step growth curves at 6 months (A) , 9 months (B) ,12 months (C) and for re-exposure experiment (D). Bars show mean ±SD (n=3).

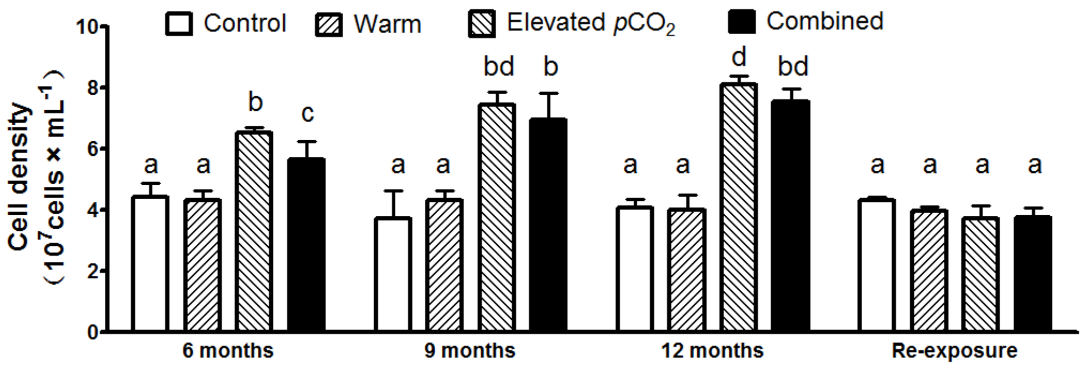

Supplementary Figure 2. The cell density of Plectonema boryanum for each treatment. Bars show mean +/- SD (n=3).

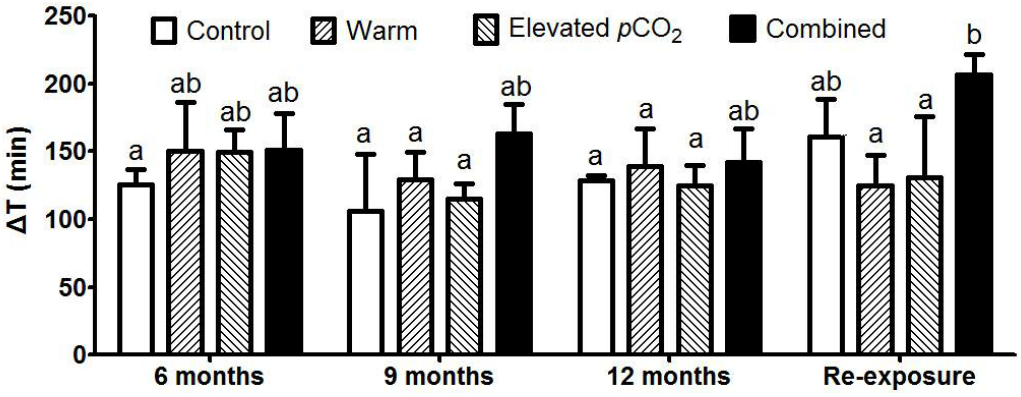

Supplementary Figure 3. The difference between the lytic cycle and latent period (Δt). Bars show mean ± SD (n=3).

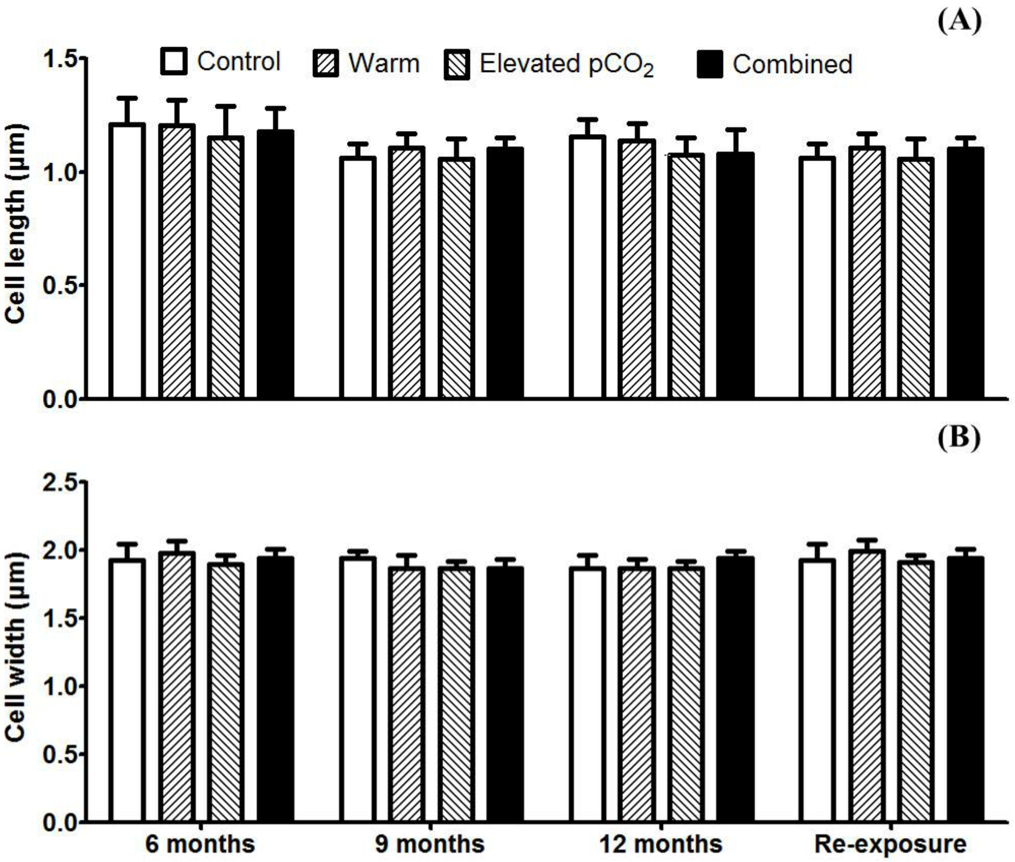

Supplementary Figure 4. Host cell length (A) and width (B) for each treatment. Bars show mean ± SD (n=3).
